# One-Step Fabrication of Hot-Water-Repellent Surfaces

**DOI:** 10.3390/biomimetics7020072

**Published:** 2022-06-04

**Authors:** Yahua Liu, Zhixin Feng, Haiyang Zhan, Wenna Ge, Yuhang Xia, Junqiu Zhang, Shile Feng

**Affiliations:** 1 Key Laboratory for Precision & Non-traditional Machining Technology of Ministry of Education, Dalian University of Technology, Dalian 116024, China; yahualiu@dlut.edu.cn (Y.L.); fzx0503@mail.dlut.edu.cn (Z.F.); zhanhy123@mail.dlut.edu.cn (H.Z.); evanna@mail.dlut.edu.cn (W.G.); xyh2020@mail.dlut.edu.cn (Y.X.); 2 Key Laboratory of Bionic Engineering, Ministry of Education, Jilin University, Changchun 130022, China; junqiuzhang@jlu.edu.cn

**Keywords:** superhydrophobic surfaces, hot-water repellency, one-step fabrication, self-cleaning

## Abstract

Hot-water repellency is of great challenge on traditional superhydrophobic surfaces due to the condensation of tiny droplets within the cavities of surface textures, which builds liquid bridges to connect the substrate and hot water and thus destroys the surface water-repellence performance. For the unique structural features and scales, current approaches to fabricate surfaces with hot-water repellency are always complicated and modified by fluorocarbon. Here, we propose a facile and fluorine-free one-step vapor-deposition method for fabricating excellent hot-water-repellent surfaces, which at room temperature even repel water droplets of temperature up to 90 °C as well as other normal-temperature droplets with surface tension higher than 48.4 mN/m. We show that whether the unique hot-water repellency is achieved depends on a trade-off between the solid–liquid contact time and hot-vapor condensation time, which determines the probability of formation of liquid bridges between the substrate and hot-water. Moreover, the designed surfaces exhibit excellent self-cleaning performance in some specific situations, such as oil medium, hot water and condensation environments. We envision that this facile and fluorine-free strategy for fabricating excellent hot-water-repellent surfaces could be valuable in popularizing their practical applications.

## 1. Introduction

Superhydrophobic surfaces inspired by natural species have attracted extensive attention in both academic and industrial fields [1,2,3,4,5]. Recent efforts have demonstrated that superhydrophobic surfaces possess two basic characteristics, i.e., micro- and nanostructures and low surface energy [6,7,8]. In these studies, water droplets at normal temperature are usually used to evaluate the superhydrophobicity of surfaces at room temperature. However, when hot-water droplets contact these superhydrophobic surfaces, the repellency performance may be destroyed. This greatly affects the application of superhydrophobic surface in the fields of water purification [9], scald-prevention clothing [10] and industrial heat exchange [11].

Studies have shown that hot-repellency performance may be destroyed by hot water due to three aspects [10,11,12,13,14]: (1) Surface tension of water droplets decreases with the increase in temperature; (2) Surface structure and low-surface-energy materials may be destroyed by hot water; (3) Hot-water vapor condenses into tiny droplets within the cavities of surface textures, which generates a liquid bridge to connect and stick the droplet to the substrate. To address these drawbacks, functional materials with hot-water repellency have been actively explored [15,16,17,18,19]. Li et al. [20] fabricated a hot-water-repellent surface by using compact and rough structures to reduce the formation of liquid bridges between droplet and substrate. Zhang et al. [21] designed surfaces with cross-networked micro-/nanostructures to achieve hot-water repellency via fluorination modification of multiwalled carbon nanotubes. Liu et al. [22] combined a hydrothermal process and electrostatic-spinning technology to prepare a double-layer gas-layer structure consisting of cuboid array structure and micronetwork structure on conductive glass, and then modified with fluorocarbon to obtain a hot-water-repellent surface. Zhang et al. [23] fabricated a hot-water-repellent surface with a dianthus caryophyllus-like micro/nanostructure by hard anodic oxidation process and fluorination modification. However, current methods for fabricating surfaces with hot-water repellency are always complicated for their unique structural features and scales. Moreover, the surfaces are modified by fluorocarbons, whose bioaccumulation, toxicity and persistence may cause environmental problems. Therefore, it is essential to develop a facile and fluorine-free approach to fabricate functional surfaces with excellent hot-water repellency.

Here, a one-step vapor-deposition process is developed to fabricate excellent hot-water-repellent surfaces (HWRSs) without extra fluorination modification. The HWRS can repel hot-water droplets in a wide temperature domain of less than 90 °C and the corresponding mechanism for hot-water repellency is discussed. In addition, we demonstrate that the HWRS can repel various liquids at room temperature, such as acidic, alkaline, coffee, as well as achieve excellent self-cleaning performance in oil medium, hot water and condensation environments.

## 2. Materials and Methods

### 2.1. Materials

The silicone resin (195T) whose main component is polysiloxane(Si_n_O_n−1_H_2n+2_) was purchased from Shenzhen Ausbond Co., Ltd. (Shenzhen, China). The crucibles (60 × 30 × 15 mm) were purchased from Jiangsu Huida Medical Instruments Co., Ltd. (Yancheng, China). Absolute ethanol, acetone, petroleum ether, n-hexane and n-heptane were purchased from Tianjin Damao Chemical Reagent Factory. All reagents are analytical-grade and are used without further purification. The glass slides (7105, 25 × 75 × 1 mm) were purchased from Dalian Liaodong Chemical Reagent Factory.

### 2.2. Surfaces Fabrication

The HWRS was prepared by a one-step vapor-deposition method. Specifically, silicone resin (~1.2 mL) was first added into a crucible, and then a glass slide, cleaned with acetone, ethanol and deionized water and dried with nitrogen, respectively, was placed above the crucible at a height of 10 mm (Figure S1). Subsequently, the crucible was put into a muffle furnace with a temperature sensor (SX2-4-10, Foshan Liedong Electric Co., Ltd., Foshan, China, Figure S2). The heating temperatures were varied from 300 °C to 450 °C, respectively, and the heating time was fixed at 3 h.

### 2.3. Characterizations of Fabricated Surfaces

The morphology of the fabricated surfaces was characterized by a scanning electron microscope (SEM, SUPRA 55 SAPPHIRE, Oberkochen, Germany). The surface chemical composition of fabricated surfaces was characterized by Fourier transform infrared spectroscopy (FTIR, ThermoFisher, Waltham, Massachusetts, USA). An OCA25 system (Dataphysics GmbH, Filderstadt, Germany) was employed to measure the static contact angle *θ* and sliding angle *θ*_s_ of droplets with volume of ~5 µL. The temperature of the hot droplet reported in this paper is the temperature of the liquid in the syringe. The temperature was measured by K-type thermocouples (Omega, 5TC-GG-K-36-72, Shanghai, China). The wettability of hot-water droplets was measured after the droplets stood on the surface for ~1 min to ensure complete condensation and the formation of liquid bridges [11]. For little change of contact angle between hot-water droplet and the HWRS in 1 min (Figure S3 and Movie S1), this test method can reflect the true wettability performance of hot-water droplets. The droplet temperature drops rapidly, to about room temperature, in ~1 min (Figure S4). All the data were determined by averaging five individual measurements of three samples. The three-dimensional topography of the surface was measured by a three-dimensional noncontact surface profiler (ZYGO, NV5022, Middlefield, CT, USA).

### 2.4. Hot-Water-Droplet Impacting Experiments

Hot-water-droplet impact experiments were conducted at temperature of 22 °C and relative humidity of 52%. In the experimental process, the target surface was placed on a horizontal adjustment stage. Droplets of radius ~1.5 mm at different temperatures (22, 60 and 90 °C) were released at a flow rate of 200 μL/min from a syringe pump and heated by a digital temperature-control heating belt. The temperature of the hot droplet reported in this paper is the temperature of the liquid in the syringe. The distance between the gravity center of drop and the target surface was adjusted to 10 mm, corresponding to a Weber number We = *ρV*^2^*R*/*γ* of 3.5 (22 °C). Here, *V* = 0.41 m/s, *R* = 1.5 mm, *ρ* =1000 kg/m^3^, and *γ* = 72.8 mN/m are the droplet impacting velocity, droplet radius, water density and the surface tension of the liquid, respectively. The drop impact dynamics was captured by a high-speed camera (Photron SA5, Tokyo, Japan) at a frame rate of 3000 fps, and investigated using ImageJ software (Version 1.46, National Institutes of Health, Madison, WI, USA). The experiment was repeated three times in each temperature, and the average value of the data was taken.

### 2.5. Self-Cleaning Tests on HWRS in Specific Situations

The self-cleaning performance of HWRS was tested in oil medium, hot water and condensation environments, respectively. The tests were conducted on three samples. Specifically, deionized water was dyed with blue food coloring (Lianyungang Xinai Food Technology Co., Ltd., Lianyungang, China) in the test. The ferric oxide (~300–500 nm, 97%, Shanghai Aladdin Biochemical Technology Co., Ltd., Shanghai, China) and soil (~60–540 µm, Figure S5) were used as contaminants in the self-cleaning experiment in oil medium and hot water as well as condensation environments, respectively. The self-cleaning process was recorded by a digital single-lens reflex camera (Canon, EOS 80D, Tokyo, Japan). In the self-cleaning test in oil medium, the HWRS was placed in a petri dish, part of the HWRS was immersed in oil, and part was exposed to air. The droplets were dripped onto the surface through a glue-tip dropper. In self-cleaning test through hot water, the HWRS was placed on a stage with inclined angle of 30°, and water droplets at temperature of 50 °C were released through a syringe drop by drop. Here, a thermal imager (Fotric 288, Shanghai, China) was used to record self-cleaning process from the side view. For self-cleaning test in condensation environment, at room temperature about 22 °C and humidity of 62%, the temperature of the cooling platform was set to 2 °C and tilted 30°. After the surface of the cooling platform reached the set temperature, the HWRS was placed on the cooling platform. After 30 min, the self-cleaning experiment was conducted.

## 3. Results

### 3.1. Fabrication of HWRS

**Figure 1:**
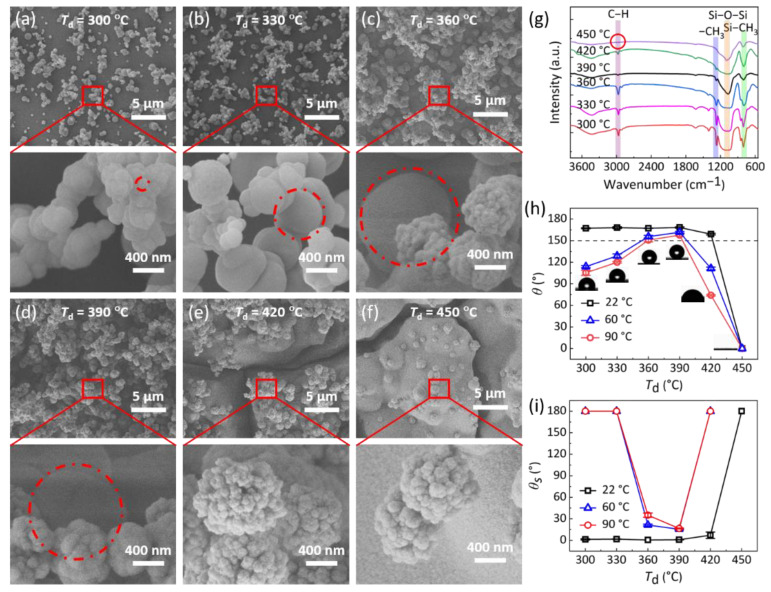
Surface morphology, chemical composition and wettability. (**a**–**f**) SEM images of surfaces fabricated at 300 °C, 330 °C, 360 °C, 390 °C, 420 °C and 450 °C, respectively. The deposition time was fixed at 3 h. (**g**) FT-IR spectra of the as-fabricated surfaces. (**h**,**i**) Contact angles (*θ*) and sliding angle (*θ*_s_) of water droplets with different temperatures on the as-prepared surfaces.

A one-step vapor-deposition approach was used to fabricate the hot-water-repellent surfaces. In the fabrication process, the deposition temperature (*T*_d_) varied from 300 °C, 330 °C, 360 °C, 390 °C, 420 °C, to 450 °C, respectively, and the deposition time was fixed at 3 h. For *T*_d_ of 300 °C and 330 °C, nanoparticles with sizes of 100–700 nm (marked with red dotted circle) were deposited on the glass substrate, while not fully covering the surface, as shown in Figure 1a,b. As *T*_d_ increased, e.g., at *T*_d_ = 360 °C and 390 °C, not only smaller-sized nanoparticles, but also large-sized microparticles of ~1 µm (marked with red dotted circle) were uniformly deposited on the substrate (Figure 1c,d). When *T*_d_ further increased, e.g., at *T*_d_ = 420 °C and 450 °C, large block structures of ~20 µm partially covered with nanoparticles were formed, which were split by cracks with size of ~2 μm (Figure 1e,f). Therefore, by controlling the deposition temperature, the surface morphology of designed surfaces can be well-tuned.

Further, FT-IR measurement was performed to investigate the chemical composition of the as-prepared surfaces (Figure 1g). The characteristic peaks at ~1086 cm^−1^, ~803 cm^−1^, ~1270 cm^−1^, and ~2968 cm^−1^ represent the asymmetric stretching vibration of the Si-O-Si bond, -CH_3_ rocking in Si-CH_3_, -CH_3_ symmetric bending in Si-CH_3_ and C-H stretching in CH_3_, respectively. Moreover, with the increase in *T*_d_, the intensity of the C-H bond decreases. For *T*_d_ = 450 °C, the C-H bond disappeared. A higher content of the C-H bond leads to a lower surface energy [24,25], indicating that the surface energy of designed surfaces can be regulated by the deposition temperature.

### 3.2. Wettability of Designed Surfaces

The wettability of the as-prepared surfaces was evaluated by water contact angle (*θ*) and sliding angle (*θ*_s_). As shown in Figure 1h, i, for droplet at temperature *T*_w_ = 22 °C, *θ* is higher than 150° and *θ*_s_ is smaller than 10° on surfaces fabricated at *T*_d_ changes from 300 °C to 420 °C, manifesting a superhydrophobic performance. This is mainly because that all these surfaces possess micro- and nanohierarchical structures and a low-surface-energy C-H bond, while surfaces fabricated at *T*_d_ of 450 °C, *θ* and *θ*_s_ are about 0° and 180°, respectively, which is because no low-surface-energy C-H bond exists on this surface (Figure 1g). For hot-water droplets at *T*_w_ of 60 °C and 90 °C, *θ* first increases and then decreases with the increase in *T*_d_, while *θ*_s_ exhibits a completely opposite trend (Figure 1h, i). Only on surfaces fabricated at *T*_d_ of 390 °C, *θ* and *θ*_s_ are higher than 150° and smaller than 16°, indicating an excellent hot-water repellency (Figure 1h,i and Figure S6). In addition, the contact angle of a high-temperature droplet (natural cooling) on the surface is still greater than 150° after 5 min (Figure S7).

The hot-water wettability behaviors on the as-prepared surfaces can be interpreted by the surface morphology. For a lower *T*_d_, e.g., 300 °C and 330 °C, the surfaces are only partially covered with nanostructures, leading to large structural gaps. When a hot-water droplet contacts the surfaces, the air in these gaps is easily replaced by tiny droplets generated by the condensation of hot-water vapor [11]. With the growth of those condensed droplets, a liquid bridge can be generated to link the hot-water droplet and substrate, transforming the solid–liquid contact state from Cassie–Baxter state to Wenzel state to destroy the water-repellency performance. When *T*_d_ rose to 360 °C, a macro- and nanohierarchical structure formed to reduce the gaps, as well as the formation probability of liquid bridges. Moreover, when *T*_d_ = 390 °C, the gaps between the structures further reduced and fewer liquid bridges formed. Therefore, the surface fabricated at *T*_d_ of 390 °C manifested excellent hot-water repellency for droplets with temperatures up to 90 °C. When *T*_d_ = 420 °C, however, because the formed larger cracks increased the generating probability of liquid bridges, the designed surface could not repel hot water effectively. Therefore, we choose the hot-water-repellent surface (HWRS) fabricated at *T*_d_ = 390 °C for further study.

### 3.3. Hot-Water-Droplet Impacting on HWRS

**Figure 2:**
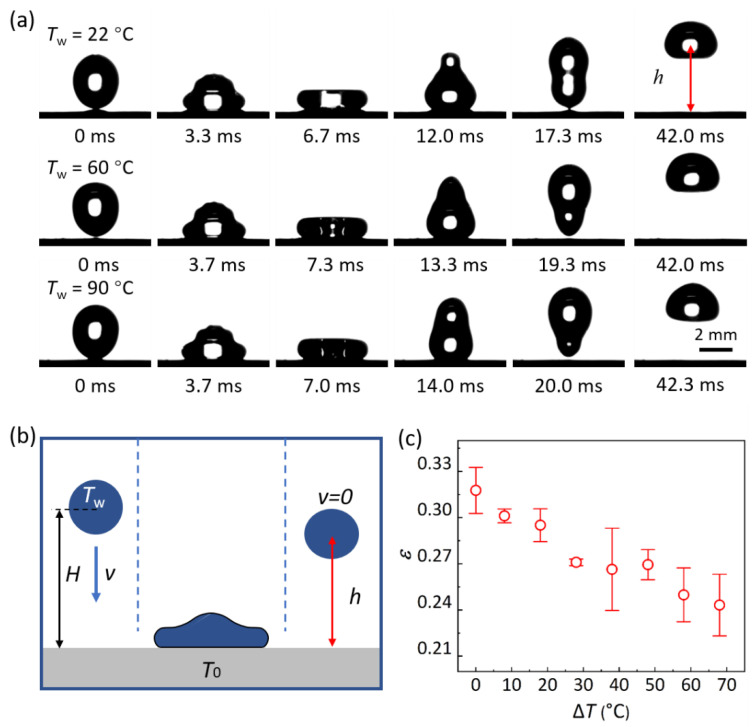
Hot-water droplets impacting on HWRS. (**a**) Snapshots showing droplets of 22 °C, 60 °C and 90 °C, respectively, impacting on the HWRS; (**b**) schematic of a hot-water droplet impacting on HWRS; (**c**) bouncing efficiency *ε* as a function of temperature difference Δ*T* = *T*_w_ *− T*_0_ between the droplet and the substrate.

A droplet impact experiment was conducted to further explore the excellence of the as-prepared surfaces. Figure 2a shows the bouncing dynamics of impacting water droplets at *T*_w_ of 22 °C, 60 °C and 90 °C on HWRS fabricated at *T*_d_ of 390 °C, of which all the droplets can bounce off the surface in less than 20 ms, while the bouncing height (*h*) (Figure 2b) decreases from 4.2 mm to 3.3 mm with the increases in *T*_w_ (Movie S2). In addition, by calculating the ratio of the long axis to the short axis of the droplet, the deviation of the droplet’s shape from sphere at the moment of impact is 1.17, 1.16 and 1.16, respectively. This result of the droplet impact indicates that the HWRS exhibits excellent hot-water repellency in the impacting process, even for droplets at *T*_w_ = 90 °C, but the kinetic-energy dissipation of the droplet becomes larger along with the increase in *T*_w_. Here, a bouncing efficiency *ε* = *E*_b_/*E*_a_ was defined to quantify the ability of a solid to repel hot water, with *E*_a_ = (2π/3)*ρR*^3^*V*^2^ and *E*_b_ = (4π/3)*ρR*^3^*g*(*h* − *R*) being the kinetic energy of the droplet before impacting and maximum potential energy after bouncing, respectively, and *g* is the gravitational acceleration. For *ε* = 0, the droplet cannot bounce off and adhere to the surface directly. For *ε* > 0, the droplet could bounce off the surface and the kinetic energy dissipation increases gradually with a decreasing *ε*. Figure 2c shows the relationship between *ε* and temperature difference (Δ*T* = *T*_w_ *−* *T*_0_) between the droplet and the substrate. It is obvious that *ε* decreased with an increasing Δ*T*, which means that the impacting drop of a higher temperature exhibits a larger kinetic-energy dissipation. Note that *ε* has a maximum of ~0.32 at normal temperature (which is higher than *ε*~0.27 in Ref. [14]), i.e., Δ*T* = 0.

## 4. Discussion

### 4.1. Mechanism of Kinetic-Energy Dissipation during Droplet Impact

To explore the mechanism of kinetic-energy dissipation in the impacting process, we analyzed the growth kinetics of the liquid bridge in the solid–liquid contact area based on the theory reported by David et al. [14,26]. When impacting on a cold superhydrophobic surface, hot water generates condensation within the cavities of surface textures, which might lead to generating a liquid bridge to connect and stick the droplet to the substrate. Note that the generation of a liquid bridge is determined by the gas condensation time (*t*_1_) and solid–liquid contact time (*t*_2_), which can be evaluated as *t*_1_~*ρh*_s_^2^/*D*∆*C*_sat_ and *t*_2_~(*ρR*^3^/*γ*)^0.5^, respectively [14]. The value of *t*_2_ is on the order of 10 ms for millimetric droplets [14]. Here, *h*_s_ is the height of surface structures, *D* is ~20 mm^2^/s is the diffusion coefficient of vapor in air, and ∆*C*_sat_ = *C*_sat_(*T*_w_) *−* *C*_sat_(*T*_0_) is the vapor-mass-concentration difference at temperatures of *T*_w_ and *T*_0_. When *t*_1_ is larger than *t*_2_, the condensed tiny droplets are smaller than the structure height *h*_s_, and thus no liquid bridge generated to link the substrate and water droplet. For the opposite condition that *t*_1_ is smaller than *t*_2_, liquid bridges generate to connect the droplet to the substrate, during which the adhesion force (*F*) can be expressed as *F ≈* 4π*R*_m_*γN*, where *R*_m_ is the maximum radius of solid–liquid interface and *N* is the probability of having a water nucleus in a cell. The energy (*E*_adh_) induced by the adhesion *F* on the radius *R*_m_ yields *E*_adh_ = 2π*γR*_m_^2^*N*. Since the viscous dissipation generated during the impact is much smaller than *E*_a_, we ignore its effect [27]. For *E*_a_ > *E*_adh_, the droplet will bounce off the surface and *E*_adh_ is equal to the kinetic-energy dissipation in the bouncing process. For *E*_a_ ≤ *E*_adh_, the droplet will never bounce, but stick to the surface directly.

**Figure 3:**
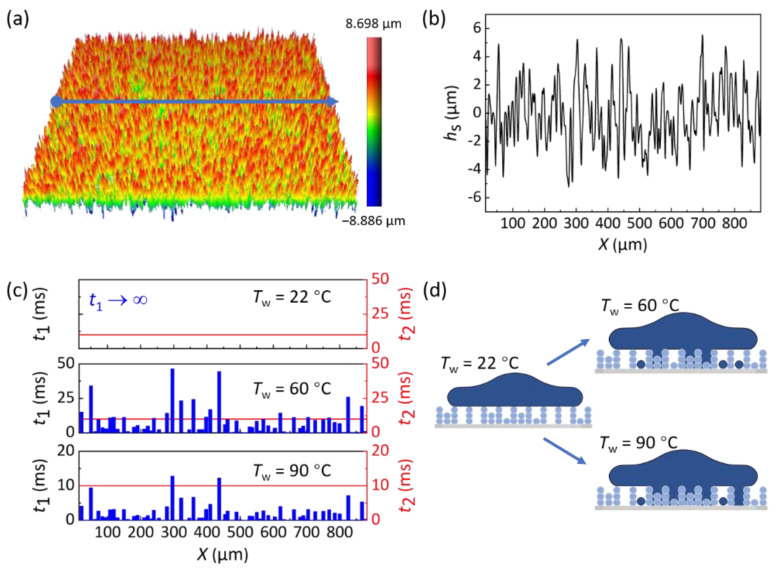
The relationship between surface textures and the formation of liquid bridges. (**a**,**b**) The texture height (*h*_s_) distribution at different locations (*X*) on the as-prepared HWRS. (**c**) The relationship between *t*_1_ and *t*_2_ on different locations of HWRS. (**d**) Schematic of the formation modes of liquid bridge at different *T*_w_.

To test this interpretation, we examined the structural height (*h*_s_) distribution at different locations (*X*) on the as-prepared HWRS via three-dimensional profile measurement (Figure 3a,b). Based on this, we obtain the relationship between *t*_1_ and *t*_2_ on different locations of HWRS (Figure 3c). For a droplet of *T*_w_ = 22 °C, ∆*C*_sat_ = 0 indicates an infinite of *t*_1_, which is far greater than *t*_2_ = 10 ms. In this case, no liquid bridges formed between the droplet and substrate, given to a maximal ε. For a droplet of *T*_w_ = 60 °C, ∆*C*_sat_ = 0.102 kg/m^3^. *t*_1_ at ~77% of surface area is smaller than *t*_2_ = 10 ms, which means that liquid bridges may generate on 77% of the surface area. For droplet of *T*_w_ = 90 °C, ∆*C*_sat_ = 0.391 kg/m^3^. *t*_1_ on the whole surface area is smaller than *t*_2_, which indicates that there is a possibility to generate liquid bridges on the whole surface. The schematics of the formation modes of liquid bridges at different *T*_w_ are shown in Figure 3d. Therefore, the generating possibility of the liquid bridge as well as the kinetic-energy dissipation increases with the increase in *T*_w_, which is consistent with the above theory.

### 4.2. Wettability of Different Liquids on HWRS

**Figure 4:**
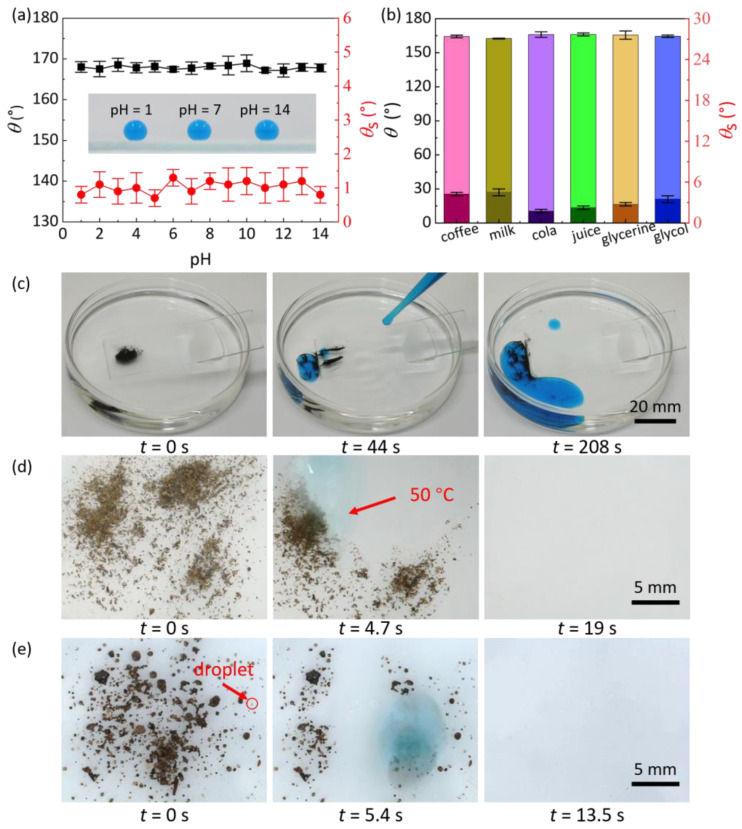
Wettability of different kinds of droplets on HWRS and self-cleaning of HWRS in specific situations, such as oil medium, hot water and condensation environments. (**a**) The relationship between *θ* and *θ*_s_ and the PH value of liquid. (**b**) The values of *θ* and *θ*_s_ of various kinds of droplets on HWRS. (**c**) Self-cleaning behavior of the HWRS immersed into the petroleum ether. (**d**) Self-cleaning behavior of the HWRS through hot water. (**e**) Self-cleaning behavior of the HWRS by falling water in condensing environment.

To demonstrate the excellent repellency of HWRS, the wettability of different liquids on the surfaces was investigated. As shown in Figure 4a, for droplets with pH value ranging from 1 to 14, the *θ* and *θ*_s_ are always higher than 165° and lower than 3°, respectively, indicating that HWRS has excellent repellency to acid-base liquid. Moreover, HWRS can repel hot acids and hot bases (Figure S8). In addition, HWRS exhibits excellent antifouling performance that various real-life liquids, including coffee, drink, milk and juice are easy to roll off with *θ* and *θ*_s_ higher than 162° and lower than 5° (Figure 4b), which is held even for low-surface-tension liquid of glycerol (*γ* = 63.3 mN/m at 20 °C) and ethylene glycol *γ* = 48.4 mN/m at 20 °C). As example, the antifouling performance of HWRS for cola is shown in Figure S9, and a low adhesion was observed between the liquid drop and the substrate (Figure S10).

### 4.3. Self-Cleaning of HWRS by Falling Water in Specific Situations

Superhydrophobic surfaces manifest excellent self-cleaning performance in general conditions [25,28,29,30], while they are subject to loss of function in specific situations, such as in oil medium, hot water and condensation environments. That is because oil, hot-water droplets and condensed droplets can penetrate the textures, and hence destroying the surface superhydrophobicity and self-cleaning properties. We first tested the self-cleaning performance of HWRS in oil medium. As shown in Figure S11, the HWRS exhibits excellent superhydrophobicity in mediums of petroleum ether, n-hexane and n-heptane with *θ* of 165°, 163°, 162° and *θ*_s_ of 0.5°, 0.5°, 4°, respectively, and experiment reveals the self-cleaning ability of the surface in oil medium that the sands on HWRS are easily taken away by sliding water droplets (Figure 4c and Movie S3). The self-cleaning property of HWRS was further verified through sliding hot-water droplets (~50 °C), that the contaminated powders could be easily removed by hot-water droplets (Figure 4d and Figure S12, Movie S4). Moreover, though condensed tiny droplets are generated on HWRS (first image in Figure 4e) in condensation environments, the superhydrophobicity of the surface was not destroyed by tiny droplets; the contaminated surface was quickly cleaned by continuous water flow (Figure 4e and Movie S5). The above experiments demonstrated excellent self-cleaning performance of HWRS by falling water in oil medium, hot water and condensation environments.

## 5. Conclusions

In conclusion, a facile and fluorine-free one-step vapor-deposition method was developed for fabricating excellent hot-water-repellent surfaces. The surface structure, chemical composition and wettability could be controlled by varying the deposition temperature *T*_d_. When *T*_d_ = 390 °C, the as-prepared surfaces manifest excellent repellency to hot-water droplets of temperature up to 90 °C and various liquids at room temperature. By analyzing the relationship between surface structure and the appearing proportion of liquid bridges between substrate and hot water, we revealed the mechanism of kinetic-energy dissipation in the impacting process. Moreover, the HWRS can achieve excellent self-cleaning performance in oil medium, hot water and condensation environments. We envision that the excellent hot-water-repellent surfaces fabricated through the facile and fluorine-free method will be promising in various industrial settings.

## Data Availability

Not applicable.

## Supplementary Materials

The following supporting information can be downloaded at: https://www.mdpi.com/article/10.3390/biomimetics7020072/s1, Figure S1: Schematic showing the surface-fabrication process; Figure S2: Image of muffle furnace with a temperature sensor; Figure S3: The contact angle between hot-water droplets at 90°C and HWRS in 1 min; Figure S4: The change in droplet temperature in 1 min; Figure S5: The size of the sand used for self-cleaning test; Figure S6: Optical and infrared images of high-temperature droplets on surface fabricated at *T*_d_ = 390 °C; Figure S7: The change of contact angle with the increase of time during the natural cooling of high-temperature droplets; Figure S8: The *θ* and *θ*_s_ of hot acid and hot base on the HWRS: (a) acid, (b) base; Figure S9: The antifouling test of HWRS; Figure S10: Low adhesion between HWRS and ethylene glycol droplet; Figure S11: The *θ* and *θ*_s_ of water on the HWRS immersed in oil: (a) petroleum ether, (b) n-hexane and (c) n-heptane; Figure S12: Sequential images showing the self-cleaning property of the HWRS by an infrared camera; Figure S13: The calibration of the infrared camera; Movie S1: The CA of 90 °C droplets in HWRS in 1 min; Movie S2: Bouncing dynamics of impacting water droplets at *T*_w_ of 22 °C, 60 °C and 90 °C on HWRS; Movie S3: Self-cleaning behavior of the HWRS immersed into the petroleum ether; Movie S4: Self-cleaning behavior of the HWRS through hot water; Movie S5: Self-cleaning behavior of the HWRS by falling water in condensation environment.
