# Supplementary material for: One-Step Fabrication of Hot-Water-Repellent Surfaces
Source: Biomimetics (Basel). 2022 Jun 4;7(2):72. doi: 10.3390/biomimetics7020072 (PMC9221071; doi:10.3390/biomimetics7020072)
Supplement: Supplementary file 1 [file biomimetics-07-00072-s001.zip › biomimetics-1720369-supplementary.pdf]

Article

One-step Fabrication of Hot-Water Repellent Surfaces

Supplimary Material

Yahua Liu [1,2], Zhixin Feng [1], Haiyang Zhan [1], Wenna Ge [1], Yuhang Xia [1], Junqiu Zhang [2], Shile Feng [1,*]

1 Key Laboratory for Precision & Non-traditional Machining Technology of Ministry of Education, Dalian University of Technology, Dalian 116024, China; fengshile@dlut.edu.cn
2 Key Laboratory of Bionic Engineering, Ministry of Education, Jilin University, Changchun 130022, China
* Correspondence: fengshile@dlut.edu.cn; Tel.: 18811434728

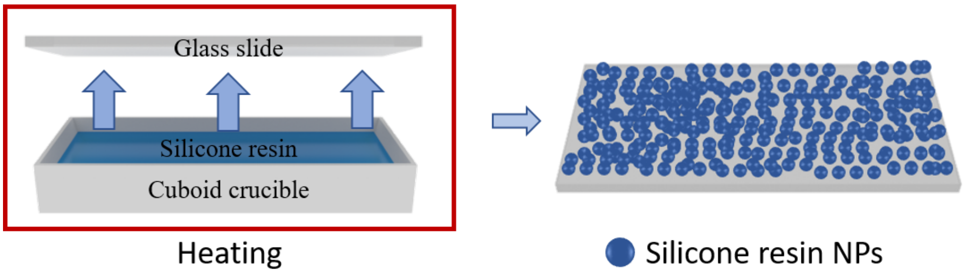

Figure S1. Schematic showing the surface fabrication process.

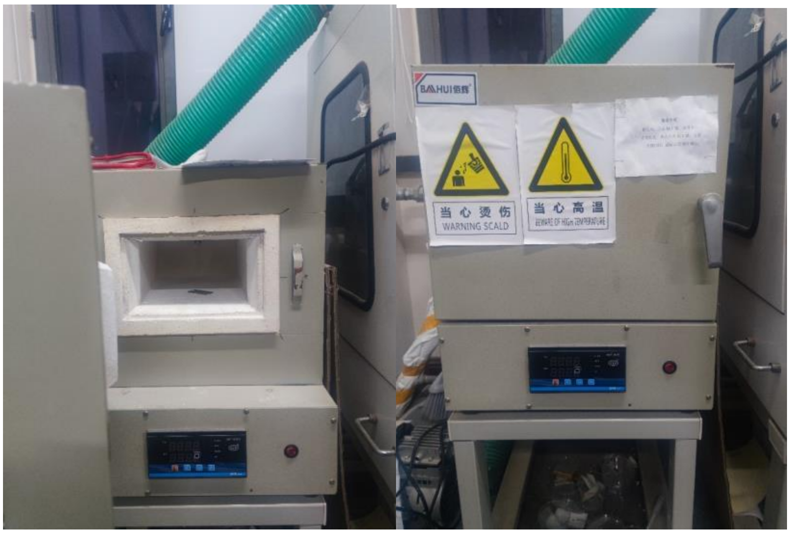

Figure S2. Image of muffle furnace with a temperature sensor.

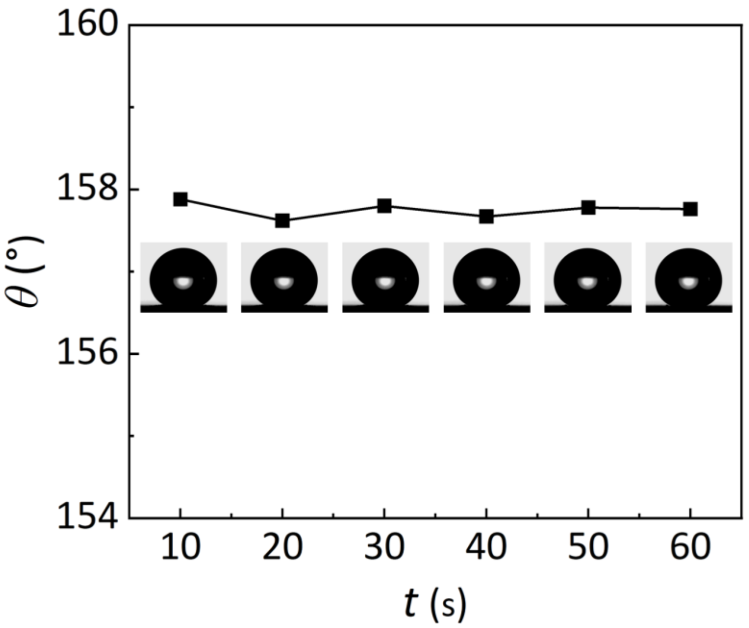

Figure S3. The contact angle between hot water droplets at 90°C and HWRS in 1 minute.

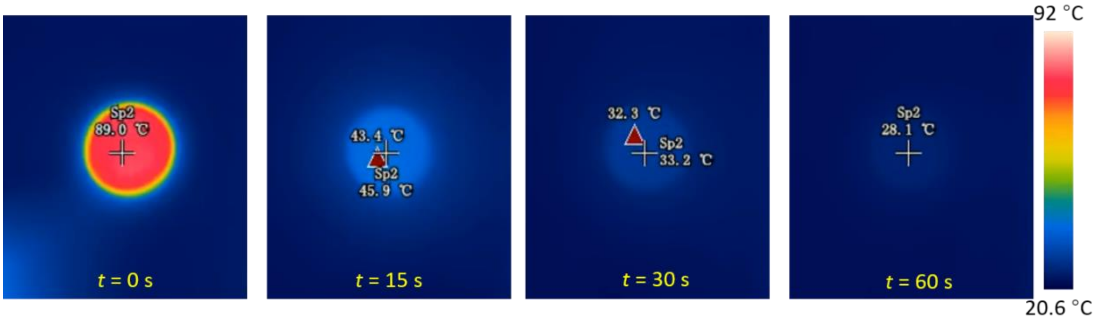

Figure S4. The change of droplet temperature in 1 min.

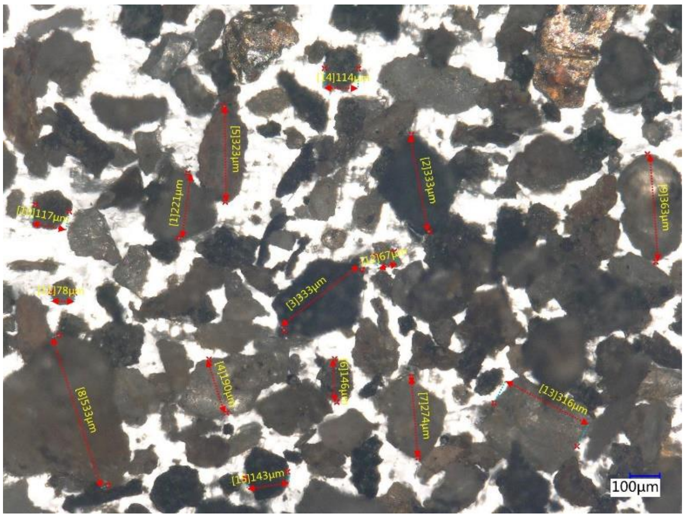

Figure S5. The size of the sand used for self-cleaning test.

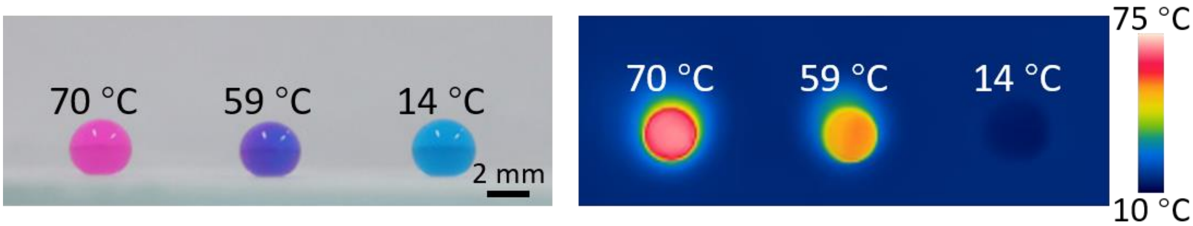

Figure S6. Optical and infrared images of high temperature droplets on the surface fabricated at T_d = 390 °C(The droplets are stained with food coloring for differentiation, and the background temperature is 18° C).

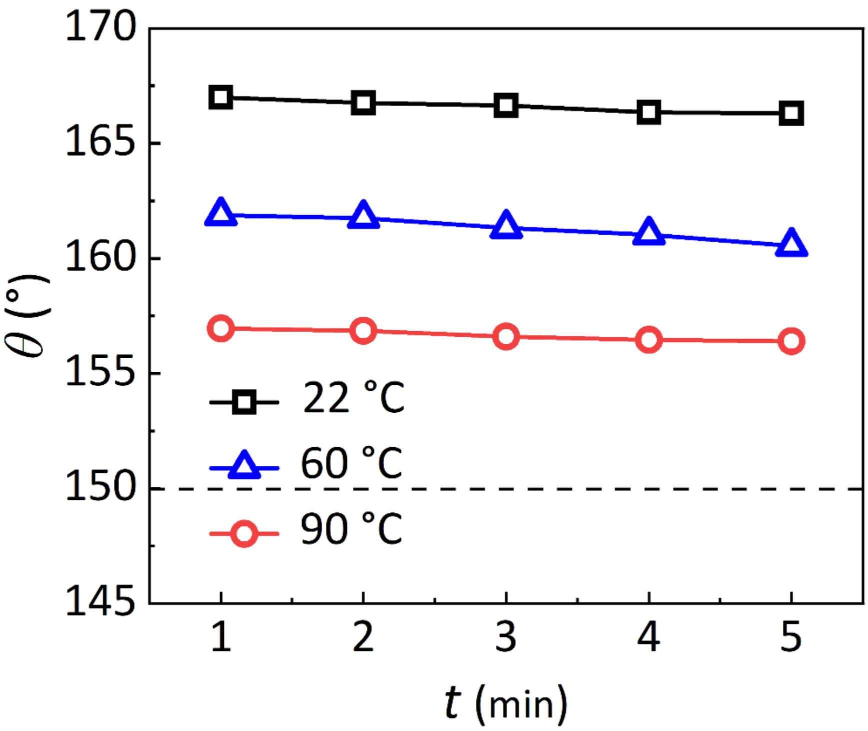

Figure S7. The change of contact angle with the increase of time during the natural cooling of high-temperature droplets.

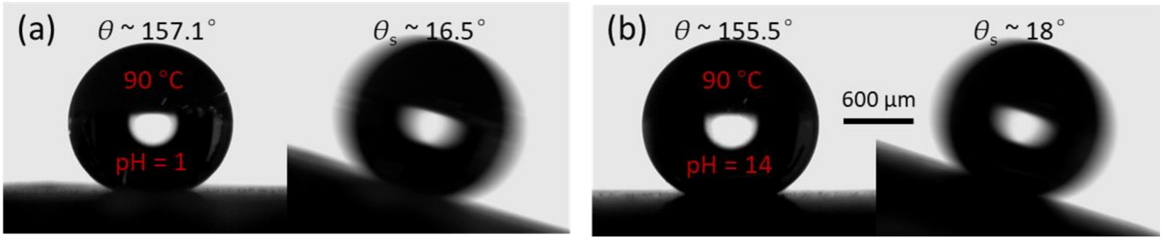

Figure S8. The θ and θ_s of hot acid and hot base on the HWRS: (a) acid, (b) base.

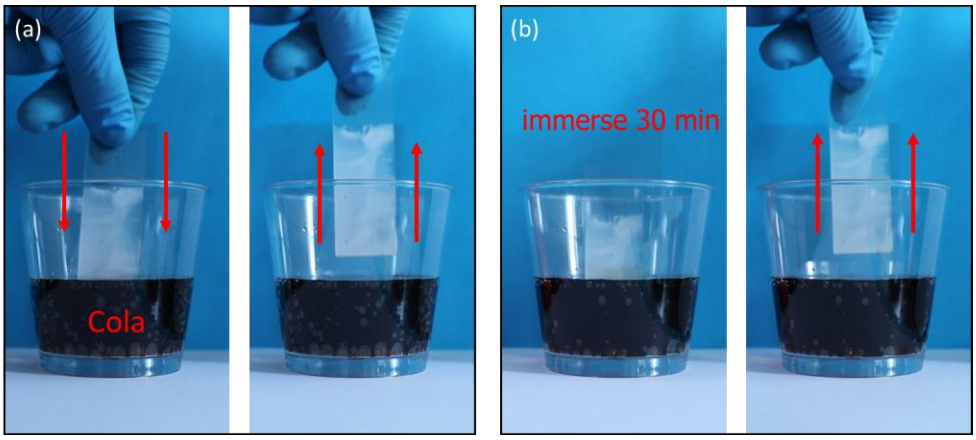

Figure S9. The antifouling test of HWRS: (a) immersed in cola and take out; (b) immersed in cola for 30 min and take out.

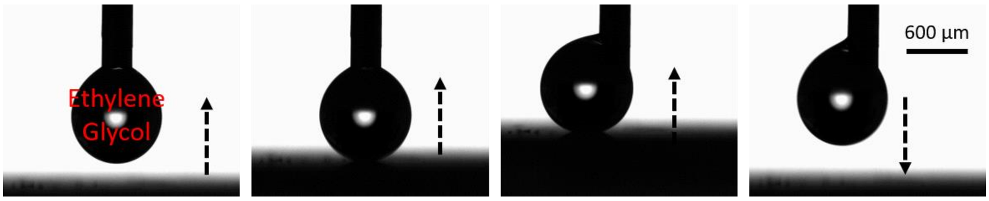

Figure S10. Low adhesion between HWRS and ethylene glycol droplet.

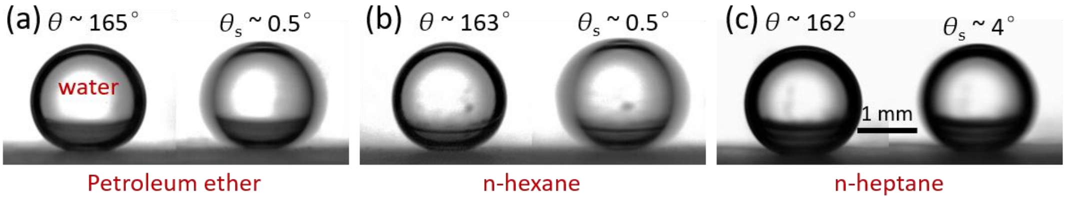

Figure S11. The θ and θ_s of water on the HWRS immersed in oil: (a) petroleum ether, (b) n-hexane, and (c) n-heptane.

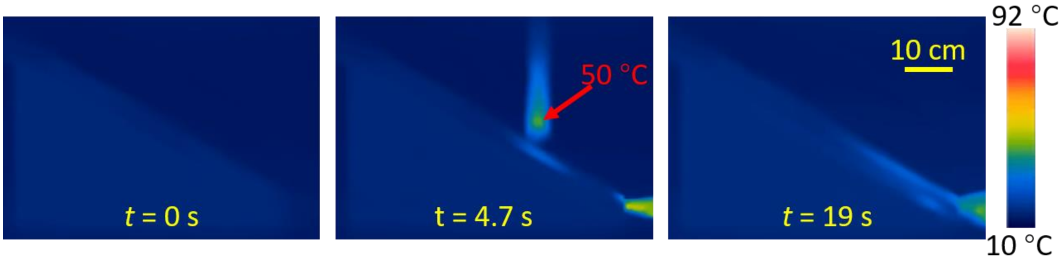

Figure S12. Sequential images showing the self-cleaning property of the HWRS by an infrared camera.

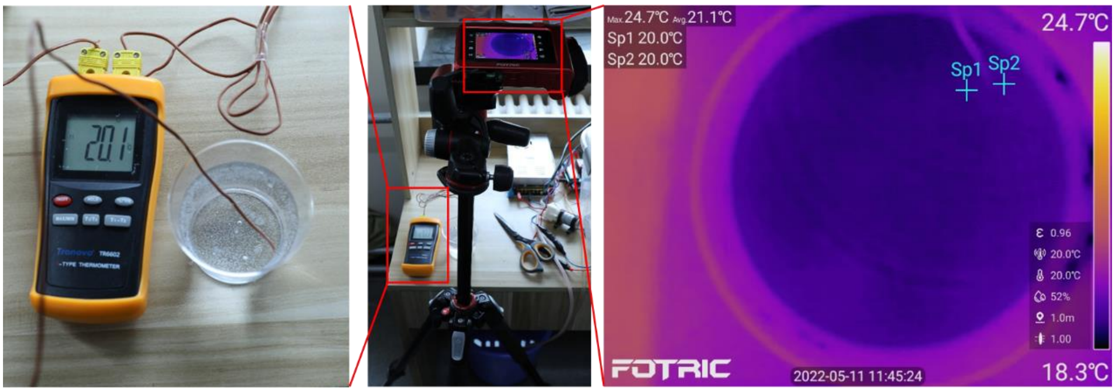

Figure S13. The calibration of the infrared camera: Firstly, the liquid temperature was measured by k-type thermocouples, and then the infrared camera was adjusted to make the temperature in the thermal imaging consistent with the measured temperature. .
